# Self-Harm History, Anxiety-Depression, Severity of Disease, and Insight Are Significantly Associated With Suicide Risk in Forensic Psychiatric Inpatients of China

**DOI:** 10.3389/fpsyt.2021.706416

**Published:** 2021-09-24

**Authors:** Huijuan Guo, Shaoling Zhong, Yuchen Yue, Ningzhi Gou, Qiaoling Sun, Xiaoxi Liang, Fanglan Wang, Juntao Lu, Qiguang Li, Jiansong Zhou, Xiaoping Wang

**Affiliations:** ^1^National Clinical Research Center for Mental Disorders, and Department of Psychiatry, The Second Xiangya Hospital of Central South University, Changsha, China; ^2^Department of Psychiatry at the Centre for Addiction and Mental Health, University of Toronto, Toronto, ON, Canada

**Keywords:** suicide, risk factors, forensic psychiatric inpatients, self-harm history, anxiety-depression, insight, severity of disease

## Abstract

**Background:** Forensic psychiatric patients have higher suicide risk than the general population. This study aimed to evaluate the extent of suicide risk and to explore the associated factors in forensic psychiatric inpatients in China.

**Methods:** We conducted a cross-sectional study from 1^st^ November, 2018 to 30^th^ January, 2019 in the Forensic Psychiatric Hospital of Hunan Province, China. Patient's information on socio-demographic, clinical, and criminological characteristics was collected. The suicidality subscale of the MINI-International Neuropsychiatric Interview (M.I.N.I.), the Brief Psychiatric Rating Scale (BPRS), and the Severity of Illness of Clinical Global Impressions Scale (CGI-SI) were used to measure present suicide risks, psychiatric symptoms, and the severity of the patient's disease, respectively. Binary logistic regression models were used to examine factors associated with suicide risk.

**Results:** Twenty-one percent (84/408) of the forensic psychiatric inpatients reported suicide risk. Logistic regression analysis suggested that self-harm history (OR:3.47, 95% confidence interval CI: 1.45–8.33), symptoms of anxiety-depression (OR:1.15, 95% CI:1.04–1.27), and more severe mental disorder (OR:1.42, 95% CI:1.08–1.87) were associated with elevated suicide risk, while insight disorder (OR:0.81, 95% CI:0.65–0.99) was related to decreasing suicide risk.

**Conclusion:** The study supplied useful clinical information to recognize high suicide risk in forensic psychiatric inpatients and may aid the development of valuable strategies for preventing and reducing suicide events.

## Introduction

Forensic psychiatric institutions typically provide high secure health services (including full range of clinical assessments and treatments) for psychiatric patients with criminal involvement. The main aim of the forensic psychiatric services is to improve the patients' psychiatric symptoms and reduce the risk of violence. The number of inpatients in the forensic psychiatric service system has been increasing in many countries (1–3). For example, the mean annual rate of increase in forensic beds was 5.7% in Austria, 5% in Germany, 4% in England, and 7% in the Netherlands per head of population between 1990 and 2006 (2, 4). Similarly, in China, the number of forensic beds increased at a rate of 1.1% between 1990 and 2009, and over 7,000 patients were detained at the present time (4, 5). In China, under criminal law, mentally ill offenders who are identified as “incapable of criminal responsibility,” and the risk assessments show that they may still pose serious harm to the public must be detained in forensic psychiatric hospitals, which are similar to maximum-security hospitals in the UK and US. These forensic psychiatric hospitals of China are funded by the local government and managed by the Public Security Bureau (4–6).

Individuals with severe mental illness have a higher suicide risk than the general population (1, 7), particularly if they exhibit severe violent behavior (8, 9). Evidence has shown that patients in forensic psychiatric wards have high rates of suicide (10). One study reported a suicide rate of 0.2% in a forensic hospital in the US, which is approximately 13 times that for all males in the general U.S. population (11). A national follow-up study carried out for more than 29 years in England and Wales showed that the suicide rate was 40 times higher for women and nearly seven times higher for men in high security hospitals than in the general population (1). Specifically, many patients remained at high risk of suicide after discharge from forensic hospitals (1). Identification of risk factors associated with suicide in forensic psychiatric inpatients is essential to screen those at high risk of suicide, which could provide some important information for the formulation and implementation of effective strategies of suicide prevention.

Suicide is a multifactorial phenomenon. Associated risk factors, including severity of mental disorder (12), self-injury history, and previous suicide attempts were identified among patients with mental illness in previous studies (13). Some evidence has shown that a high risk of suicide may be related to imprisonment, length of hospitalization, and psychiatric symptoms (14, 15). Few studies on suicide in Chinese forensic psychiatric hospitals have been conducted. Most of these studies were descriptive or had small sample sizes (16, 17). For example, our previous qualitative study conducted in a forensic psychiatric hospital showed that many long-stay patients experienced negative emotions and feelings, including loneliness, worthlessness, and hopelessness, and some reported suicidal thoughts and suicide attempts (18). However, the associated risk factors for suicide among forensic psychiatric inpatients are unknown. Therefore, it is necessary to explore the suicide risk and the independently related factors in forensic psychiatric inpatients. In this study, we focused on patients in the Hunan Provincial Forensic Psychiatric Hospital in China, and explored possible contributors to suicide risk in this group.

## Methods

### Study Population

We conducted a cross-sectional study from 1^st^ November, 2018 to 30^th^ January, 2019 in the Hunan Forensic Psychiatric Hospital, which is the only forensic psychiatric hospital in Hunan Province of China and serves more than 73.19 million people (according to the Hunan Provincial Bureau of Statistics in 2019). The hospital, located in Yueyang City, is managed by the public security system (18) and equipped with five wards, and is staffed by 13 clinicians and 21 nurses. At the time of this study, the hospital had 461 inpatients during the study.

Patients were recruited if they met the eligibility criteria: they (a) were able to communicate adequately (talk or write) and (b) could comprehend the objective of the study. The exclusion criteria were as follows: (a) refused to take part in the interview and (b) unable to talk or write. All study participants provided signed informed consent.

### Tools and Evaluation

A standard questionnaire was used to collect socio-demographic, clinical and criminological information, including gender, age, education level, residence, marital status, employment before mandatory hospitalization, history of psychiatric treatment, family history of mental disorders, and the self-harm history. Current offense type (homicide/non-homicide) were collected from official criminal records. Clinical data, such as the information on the length of stay and recent antipsychotic dosages were extracted from their medical records. Antipsychotic medication dosages were converted into the corresponding clozapine dose equivalent according to the defined daily doses (DDDs) method (19, 20).

The Brief Psychiatric Rating Scale (BPRS) was used to measure psychiatric symptoms. The most commonly used version comprises 18 items (21). Two additional items—“insight disorder and work ability” (22) were added by the Chinese Scale Cooperation Group. The BPRS has demonstrated good reliability and validity in practice in many countries, including China (23, 24). Five subscales were included: (a) anergia factors (emotional withdrawal, motor retardation, blunted affect, and disorientation), (b) anxiety-depression factors (somatic concern, anxiety, guilty feelings, and depressive mood), (c) thought disturbance factors (conceptual disorganization, grandiosity, hallucination, and unusual thought content), (d) hostile suspiciousness factors (hostility, suspiciousness, and uncooperativeness), and (e) activation factors (tension, mannerism-posturing, and excitement) (25). The factor score denotes the distribution of symptoms and the clinical characteristics of the disease. Two additional items were (X1) insight disorder, which refers to the lack of awareness of one's mental illness, mental symptoms, or abnormal words and behaviors, and (X2) impaired work ability that refers to the impact on daily work or activities (22). Clinical Global Impression severity scale (CGI-SI) was used to measure the severity of the patient's disease, which is a commonly used tool for comprehensive evaluation of severity of illness in psychiatry (26). The item uses an 8-point scoring method ranging from 0 to 7, with higher scores indicating more severe disease.

The MINI-International Neuropsychiatric Interview (M.I.N.I.), developed by Lecrubier etc. (27) was used to assess mental disorders and suicide risk. The reliability and validity of the M.I.N.I. has been established in previous reports (28). The suicidality subscale includes six items: In the past month did you (1) Think that you would be better off dead or wish you were dead (no-0, yes-1) (2) Want to harm yourself or to injure yourself (no-0, yes-2) (3) Think about suicide (no-0, yes-6) (4) Have a suicide plan (no-0, yes-10) (5) Attempt suicide (no-0, yes-10) (6) In your lifetime, have you made a suicide attempt (no-0, yes-4). The total score is calculated by the sum of the scores for the six items. A total score equal to zero is considered indicative of no suicide risk, and a total score higher than zero is regarded as indicative of suicide risk.

### Procedures

An explanation of the purpose of this study was distributed by the researchers to all patients. Patients who agreed to take part gave their written informed consent. All participants were individually interviewed face-to-face in a private meeting room of the forensic psychiatric hospital, by three trained forensic psychiatrists. The study protocol was approved by the Human Ethics Committee of the Second Xiangya Hospital, Central South University, and the authority of the Hunan Forensic Psychiatric Hospital in China.

### Statistical Analysis

We performed tests of normality on two groups (the non-suicide risk group and suicide risk group), and found that the data were consistent with the normal distribution. Continuous variables were presented as the mean ± standard deviation (SD), and categorical variables were expressed as the number of cases and percentages. Missing data were excluded from all analyses. Comparisons between the groups were performed by *t*-tests and chi-square tests. Finally, binary logistic regressions (backward: LR) were used to examine the correlated factors with elevated suicide risk. Variables with *p* ≤ 0.10 in the univariate analyses were included in the binary logistic regression models. In addition, as insight, severity of mental illness and length of stay in hospital may affect the risk of suicide, which were indicated by previous research and clinical experience, we included the above factors in the binary logistic regression model. The statistical significance level was set at 0.05 (two-tailed). We used the Statistical Package for the Social Sciences (SPSS 23.0) to perform analyses.

## Results

**Figure 1:**
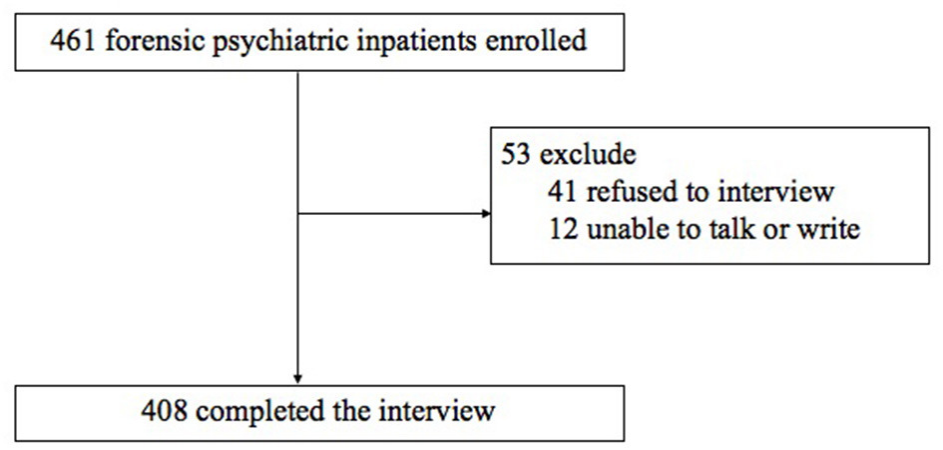
Flow chart.

A total sample of 408/461 (88.5%) patients completed the interview. Patients were excluded for the following reasons: refusal to participate in the interview (*n* = 41) and unable to talk or write (*n* = 12) (see Figure 1).

As shown in Table 1, a total of 84 inpatients (20.6%) reported apparent recent suicide risk. The mean age of the patients was 44.3 (SD = 9.1) years old, 73.8% were male, 67.5% were unmarried, and 81.8% had committed homicide, 18.2% committed other violent crime [(including assault) (*n* = 48), arson (*n* = 12), and the crime of provocation (*n* = 8)]. Approximately 91.2% of all patients were diagnosed with schizophrenia in the hospital. The average length of hospital stay was 8.30 ± 4.60 years; 78.4% of patients had stayed for over 5 years, and the longest stay was 37.11 years.

**Table 1:** Socio-demographic and criminological characteristics of the sample.

		**Non-suicide risk**		**Suicide risk**		**Statistics**		
		***n***	**%**	***n***	**%**	***χ2***	***df***	***p***
Gender (*n* = 408)						11.1	1	0.002
	Male	286	88.3	62	73.8			
	Female	38	11.7	22	26.2			
Education level (*n* = 394)						0.31	1	0.640
	Low (≤9 years)	252	81.0	65	78.3			
	High (>9 years)	59	19.0	18	21.7			
Residence (*n* = 407)						2.0	1	0.188
	Urban	36	11.1	14	16.9			
	Rural	288	88.9	69	83.1			
Unmarried (*n* = 406)						1.1	1	0.175
	No	86	26.6	27	32.5			
	Yes	237	73.4	56	67.5			
Unemployed (*n* = 392)						1.5	1	0.260
	No	135	43.7	30	42.1			
	Yes	174	56.3	53	57.9			
History of psychiatric treatment (*n* = 403)						2.7	1	0.116
	No	110	34.5	21	25.0			
	Yes	209	65.5	63	75.0			
Current type of offense (*n* = 408)						2.7	1	0.138
	Non-homicide	59	18.2	9	10.7			
	Homicide	265	81.8	75	89.3			
Family history of mental disorders (*n* = 382)						1.3	1	0.302
	No	258	84.6	61	79.2			
	Yes	47	15.4	16	20.8			
Self-harm history (*n* = 389)						7.5	1	0.010
	No	291	94.2	68	85.0			
	Yes	18	5.8	12	15.0			
		**Mean**	**SD**	**Mean**	**SD**	***t***	***df***	***p***
Age (*n* = 408)		44.3	9.1	42.0	10.1	2.0	406	0.048
Work ability (*n* = 395)		2.8	1.6	3.4	1.7	−2.7	393	0.007

*Unmarried status includes single, divorced, and widowed*.

Compared to those with no suicide risk, forensic psychiatric inpatients with suicide risk were more likely to be female (χ^2^ = 11.1, *df* = 1, *p* = 0.002), be younger (*t* = 2.0, *df* = 406, *p* = 0.048), have self-harm history (χ^2^ = 7.5, *df* = 1, *p* = 0.010), and have worse work ability (*t* = −2.7, *df* = 393, *p* = 0.007) (see Table 1). The suicide risk group had a higher recent antipsychotic dose (*t* = −2.3, *df* = 402, *p* = 0.019], and more anxiety-depression factors (*t* = −4.5, *df* = 393, *p* < 0.001) than the non-suicide risk group. Forensic psychiatric inpatients with no suicide risk were more likely to be diagnosed with schizophrenia compared to those with elevated suicide risk (see Table 2).

**Table 2:** Clinical characteristics of the sample.

	**Non-suicide risk**		**Suicide risk**		**Statistics**		
	**Mean**	**SD**	**Mean**	**SD**	***t***	***df***	***p***
Insight (*n* = 395)	4.1	2.3	3.8	2.0	1.3	393	0.183
CGI-SI(*n* = 388)	4.3	1.6	4.6	1.6	−1.5	386	0.123
Length of stay (*n* = 405)	8.35	4.3	8.09	5.1	0.5	403	0.641
Recent antipsychotic dose (*n* = 404)	248.5	135	288.7	149.8	−2.3	402	0.019
BPRS (*n* = 395)							
Anxiety-depression	5.3	2.2	6.8	3.7	−4.5	393	<0.001
Anergia	8	4	8.5	3.8	−0.9	393	0.354
Thought disturbance	6.9	3.9	7.8	4.3	−1.8	393	0.079
Activation	3.4	1	3.6	1.2	−1.5	393	0.133
Hostile suspiciousness	5.3	3.2	5.5	3.1	−0.5	393	0.650
	***n***	**%**	***n***	**%**	***χ2***	***df***	***p***
Diagnoses (*n* = 408)					20.5	7	0.005
Schizophrenia	302	93.2	70	83.3			
Non-schizophrenia	22	7.8	14	16.7			

*BPRS, The Brief Psychiatric Rating Scale; non-schizophrenia disorders include depression (n = 11), epileptic mental disorders due to epilepsy (n = 10), bipolar disorder (n = 8), schizoaffective disorder (n = 2), brain organic mental disorders (n = 2), mental disorders caused by psychoactive substances (n = 2), mental retardation (n = 1)*.

Multivariable analyses revealed that after controlled the social-demographic confounders (gender and age), self-harm history (OR = 3.47, 95% confidence interval CI:1.45–8.33), symptoms of anxiety-depression (OR = 1.15, 95% CI:1.04–1.27), and more severe mental disorder (OR = 1.42, 95% CI:1.08–1.87) were associated with elevated suicide risk, while insight disorder (OR:0.81, 95% CI:0.65–0.99) was related to decreasing suicide risk (see Table 3).

**Table 3:** Factors associated with suicide risk among forensic psychiatric inpatients (Binary logistic regression model).

**Variables**	**Unadjusted**			Adjusted^*^		
	**OR**	**95%CI**	***P*-value**	**aOR**	**95%CI**	***P*-value**
Self-harm history	4.06	1.75–9.41	0.001	3.47	1.45–8.33	0.005
Insight	0.77	0.63–0.95	0.013	0.81	0.65–0.99	0.049
Anxiety-depression of BPRS	1.15	1.05–1.26	0.004	1.15	1.04–1.27	0.005
CGI-SI	1.43	1.10–1.86	0.008	1.42	1.08–1.87	0.012

* *Controlled age and gender; OR, odds ratio; 95% CI, 95% confidence interval*.

## Discussion

To our knowledge, this is the first study to survey suicide risk and the independent contributions of socio-demographic, criminal, and clinical risk factors correlated with elevated suicide risk in forensic psychiatric inpatients in China. Our study found that one-fifth of inpatients in the forensic psychiatric system reported recent suicide risk. Self-harm history, symptoms of anxiety-depression, and more severe mental illness were associated with elevated suicide risk, while insight disorder was related to decreasing suicide risk. These findings can provide some useful information that may aid in the identification of high suicide risk in forensic psychiatric inpatients.

The rate of suicide risk (20.6%) in forensic psychiatric inpatients is comparable with findings in previous studies on patients with schizophrenia (ranging from 18 to 55%) (29–31). However, due to the differing methodologies or definitions of suicide risk in published studies, it is impossible to make an objective comparison. In general, the rate of suicide risk in forensic psychiatric inpatients is relatively high, which is a problem that deserves more attention. In particular few hospitals meet to pay attention to suicide risk and they also lack corresponding tools or guidelines for suicide risk assessment and management (32). In the present study, we found that more severe mental disease was associated with an elevated risk of suicide. A potential explanation is that people with severe mental illness may be with suicidal-related psychiatric symptoms, such as commanded auditory hallucinations that lead to suicidal ideation and behaviors, or disturbing emotions, as indicated by previous research (12).

The study also found that good insight was associated with a higher risk of suicide, similar to previous studies (33–35). Insight is defined as patients' “ability to recognize their own mental health status” and includes three dimensions: awareness of having psychotic symptoms, compliance with treatment, and views on social consequences such as hospitalization or unemployment due to mental disorder (36, 37). This is an important concept in clinical settings. Patients with mental disorders who have regained insight and are in a stable condition may be discharged from general psychiatric hospitals but cannot be released from forensic psychiatric hospitals because of the legal procedures, public safety, and subsequent supervision and other issues (38). In addition, there are no legal standards or rules for the length of incarceration. Many patients in despair of ever being discharged from the hospital, become more anxious and depressed, and even may generate feelings of hopelessness, which may increase their risk of suicide. Previous studies have reported that the phenomena of self-reproach, guilt, and self-stigmatization are common (39, 40). These factors were reported to be associated with an increased risk for suicidal behavior (41, 42). With the recovery of insight, many patients develop have a certain understanding of their diseases and the crimes they committed. Considering that all the patients committed violent crimes and most of them killed their relatives or friends, those with good insight may be more likely to feel guilt and regret for their previous behavior than those with poor insight. Some patients may feel inferior because of their mental disorder; they are worried about the disease recurring and the recurrence of violent behaviors and blame themselves for all their mistakes. Some patients may feel that life is hopeless or meaningless and even choose to end their life. All of these problems may increase their risk of suicide.

Our findings indicated that anxiety-depression was associated with elevated suicide risk, which was consistent with our clinical experience and previous research (12, 43, 44). Anxiety-depression is common in forensic psychiatric inpatients. Due to their long stay in a forensic psychiatric hospital with little freedom, many patients become worried, fearful and overly concerned about their current and future situation. Some may also feel sad, depressed, or helpless. When these negative emotions appear, they may struggle to deal with them, and may be unwilling to seek help because of fear that reporting truthfully will affect their discharge process. This may lead to worsening moods and sometimes to extreme events such as self-injury. This is another finding in the research: patients with a history of self-harm may be at higher risk of suicide, replicating the frequently reported connection between previous self-harm behavior and suicide risk in patients with mental disorders (45–47). In summary, patients indulging in negative thoughts and who are unable to defuse from them may demonstrate elevated suicide risk (41, 42, 48, 49).

Therefore, it is urgent to strengthen the assessment and intervention of suicide risk for forensic psychiatric inpatients. Strategies to reduce suicide risk in forensic psychiatric hospitals should include attention to several factors. First, some meaningful suggestions may include paying more attention to assessing the patient's mood, and implementing targeted practical rehabilitation treatment, which can help to protect the human rights and quality of life of these inpatients. For example, when a patient shows major depressive symptoms, appropriate antidepressants should be given to improve the mood. If necessary, Modified Electra-Convulsive Therapy (MECT) also can be considered. Second, many more health education activities should be developed and implemented. For people in need, psychological treatment such as cognitive behavioral therapy (50), dialectical behavioral therapy (51), or mindfulness-based stress reduction (52) can be advocated. In addition, for those who have close family relationships, staff should encourage patients to communicate with family members to obtain more support and arrange more meetings between the patients and their families. For those who have lost family relationships, facilities should strengthen the support they receive from society, giving them more confidence, and encouragement. Third, future work should also focus on improving the quality of forensic mental health services. On the one hand, improving medical resources and increasing the number of forensic psychiatric beds should be considered, as these steps would enable more “incapable of criminal responsibility patients” to receive corresponding restorative treatment. On the other hand, it is necessary to implement practical discharge procedures and implementation rules at the legal level. It is also necessary to establish a conversion mechanism for circulation among forensic psychiatric hospitals, general psychiatric hospitals, and community-based outpatient services based on the severity of illness and the safety risk. Therefore, this not only would allow patients to obtain the least restricted services but would also ensure the treatment of the disease, as patient care and public safety need not be mutually exclusive.

## Limitations

There are some study limitations that should be mentioned. First, the sample was recruited only from the Forensic Psychiatric Hospital of Hunan Province which is not representative of the population in China. Second, because of the cross-sectional study design, it was not possible to establish a causal relationship between the elevated suicide risk and identified correlates. These factors need to be verified in further cohort studies. Third, the definition of suicide risk was based on patients' self-report during interviews. Suicide risk might therefore have been underestimated as participants may hide their true thoughts and feelings. Finally, we did not investigate the combination of personality disorders in these patients although previous evidence indicated that personality disorder was less common in patients with severe mental disorders in China than in Western countries (53).

## Conclusions

In conclusion, 20.6% of patients in the forensic psychiatric hospital were reported to have recent suicide risk. The independent associated factors were self-harm history, anxiety-depression, good insight, and more severe mental illness. Given complexities entailed in reducing the rate of suicide risk and providing effective treatment for patients with high suicide risk, further investigation is needed. The results of this study can supply some useful information for suicide prevention or intervention and may help to establish more humane and engaging mental health services.

## Data Availability Statement

The raw data supporting the conclusions of this article will be made available by the corresponding authors, without undue reservation.

## Ethics Statement

The studies involving human participants were reviewed and approved by he Human Ethics Committee of the Second Xiangya Hospital, Central South University. The patients/participants provided their written informed consent to participate in this study.

## Author Contributions

HG: investigation, data curation, formal analysis, writing—original draft, writing—review, and editing. SZ: investigation, data curation, and formal analysis. YY, NG, and QS: investigation, resources, and validation. XL and FW: visualization, writing—review, and editing. JL: investigation and resources. QL: methodology, investigation, and resources. XW and JZ: writing and revising the draft and supervision. All authors read and approved the final manuscript.

## Funding

This work was supported and funded by the Science and Technology Program of Hunan Province (No. 2018SK2133) and the Hunan Innovative Province Construction Project (No. 2019SK2334).

## Conflict of Interest

The authors declare that the research was conducted in the absence of any commercial or financial relationships that could be construed as a potential conflict of interest.

## Publisher's Note

All claims expressed in this article are solely those of the authors and do not necessarily represent those of their affiliated organizations, or those of the publisher, the editors and the reviewers. Any product that may be evaluated in this article, or claim that may be made by its manufacturer, is not guaranteed or endorsed by the publisher.
